# Nearest Template Prediction: A Single-Sample-Based Flexible Class Prediction with Confidence Assessment

**DOI:** 10.1371/journal.pone.0015543

**Published:** 2010-11-23

**Authors:** Yujin Hoshida

**Affiliations:** Cancer Program, Broad Institute of Massachusetts Institute of Technology and Harvard University, Cambridge, Massachusetts, United States of America; Duke-National University of Singapore Graduate Medical School, Singapore

## Abstract

Gene-expression signature-based disease classification and clinical outcome prediction has not been widely introduced in clinical medicine as initially expected, mainly due to the lack of extensive validation needed for its clinical deployment. Obstacles include variable measurement in microarray assay, inconsistent assay platform, analytical requirement for comparable pair of training and test datasets, etc. Furthermore, as medical device helping clinical decision making, the prediction needs to be made for each single patient with a measure of its reliability. To address these issues, there is a need for flexible prediction method less sensitive to difference in experimental and analytical conditions, applicable to each single patient, and providing measure of prediction confidence. The nearest template prediction (NTP) method provides a convenient way to make class prediction with assessment of prediction confidence computed in each single patient's gene-expression data using only a list of signature genes and a test dataset. We demonstrate that the method can be flexibly applied to cross-platform, cross-species, and multiclass predictions without any optimization of analysis parameters.

## Introduction

It has been nearly a decade since genome-wide expression profiling was applied on clinical specimens with a great expectation for its potential to fish out disease-related genes as diagnostic biomarkers and/or therapeutic targets in comprehensive and unbiased manner [1]. Many studies have subsequently reported sets of coordinately dysregulated genes, i.e., gene-expression signature, correlated with clinical phenotype of interest. Some studies also have shown that the signature could be more sensitive than traditional histological assessment in monitoring biological status of diseased tissue [2]. Accompanying these studies, consensus on key issues of study design and analysis protocol emerged [3], [4] However, despite these efforts, most of these signatures have not yet been introduced into clinical practice.

As a medical device utilized for clinical diagnostic and prognostic prediction, the signatures need to be intensively evaluated before their clinical deployment. However, this critical process has been hampered by the following obstacles. First, it is widely recognized that the measurement in gene-expression microarray could vary across experimental conditions and assay platforms [5], [6], and it is still uncertain what the optimal and sustainable assay platform is given the rapid genomics technology development. Second, analytical restrictions such as requirements for comparable pair of training and test datasets, specific analytic algorithm and parameters used in the initial study, etc., often preclude the opportunity of preclinical assessment of the signature. Third, the signatures are often defined based on various types of experimental techniques applied on variety of biomolecules or even biological knowledge, which could be a valuable source of potential biomarker. However, there is no established way to utilized them for prediction analysis to estimate their potential value in clinical practice. Furthermore, as a tool to help physicians' clinical decision making, the prediction method should be applicable to each single patient, although this is overlooked in most of existing prediction methods, which were designed for a dataset consisting of multiple samples [7]. In addition, it is ideal that each prediction is accompanied with a measure of confidence to enable more reliable clinical decision making.

Nearest template prediction (NTP) is a method designed to address these issues, without requiring corresponding training dataset. It has been successfully applied for gene-expression-based clinical classification and outcome prediction [2], [8], [9]. In this article, we describe detailed methodology of NTP and its performance in comparison with other commonly used prediction methods to highlight its advantage.

## Results

### Overview of Nearest Template Prediction (NTP)

**Figure 1:**
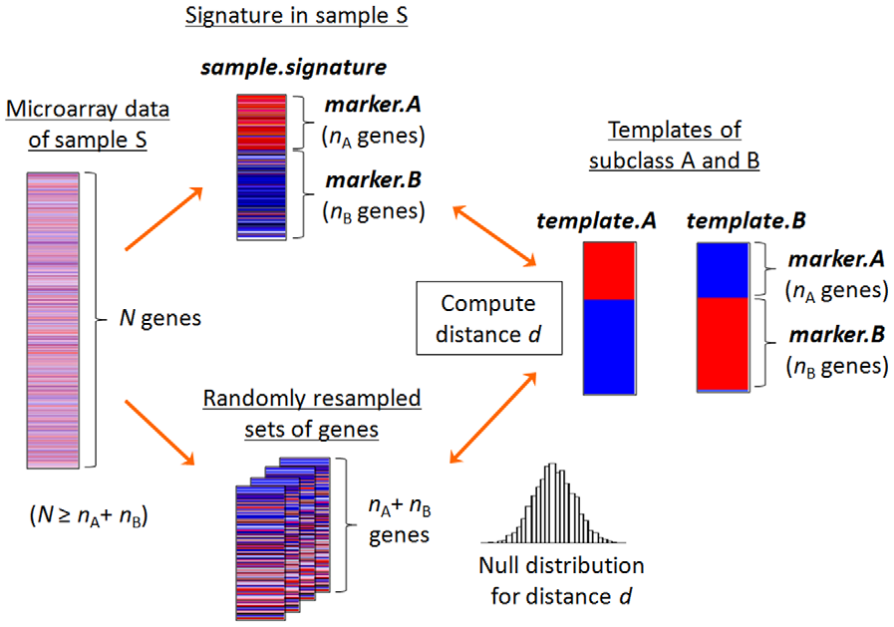
Methodology of Nearest Template Prediction (NTP). Based on predetermined gene signature of subclasses A and B including *n*_A_ over-expressed genes in subclass A (*marker.A*) and *n*_B_ over-expressed genes in subclass B (*marker.B*), *template.A* and *template.B* are defined as representative expression pattern of the signature genes for each subclass (Templates of subclass A and B). From microarray data measuring *N* genes in sample S (Microarray data of sample S), the *n*_A_+*n*_B_ signature genes are extracted (Signature in sample S: *sample.signature*), and its proximity to the templates is evaluated by calculating distance *d*. The label of closer template is assigned as a prediction for sample S, and its significance is estimated as a nominal p-value based on a null distribution for *d* generated by randomly resampling *n*_A_+*n*_B_ genes from the *N* genes 1,000 times. Red and blue colors in heatmaps indicate high and low gene expression, respectively.

Diagnostic and/or prognostic genomic signature is usually a set of genes coherently over- or under-expressed in patients with a certain phenotype of interest, or combination of these sets of genes, and assumed to indicate ON (or up) or OFF (or down) of relevant biological functions. The major task of gene-expression signature-based class prediction method would be simply to capture the presence or absence of these patterns in each sample, rather than recapitulating complex combinatorial pattern of the signature gene expression. NTP is a simple, hence flexible, nearest neighbor-based method designed to capture such information. It requires only a list of signature genes and a dataset to be tested. Methodological details are described in the Materials and Methods section and **Figure 1**. Here we overview the key steps:

### Step 1: Define representative expression pattern of the signature genes (template)

For simplicity, suppose the case of prediction of 2 subclasses, A and B. The signature genes consist of *n_A_* (*marker.A*) and *n_B_* (*marker.B*) over-expressed marker genes in subclass A and B, respectively. In a hypothetical subclass A sample, it is supposed that *marker.A* are coherently over-expressed and *marker.B* are under-expressed. If the expression level of each signature gene is standardized, genes in *marker*.A are expected to show uniformly high, and genes in *marker*.B are expected to show uniformly low expression. The expression pattern of the signature in a subclass B sample is similarly assumed. These expression patterns of hypothetical samples representing the subclasses are used as templates for the prediction. Specifically, a value of 1 is assigned to *marker.A* and −1 is assigned to *marker.B* for the template of subclass A (*template*.A). The template of subclass B (*template*.B) is similarly defined.

### Step 2: Find nearest template to assign a prediction label to a test sample

Suppose *N* genes are measured in a microarray experiment for a test sample S (*N*≥*n_A_*+*n_B_*). Expression levels of the *n_A_*+*n_B_* signature genes (*marker.A* and *marker.B*) are extracted from the *N* genes (*sample.signature*). Subsequently, proximity of *sample.signature* to *template.A* or *template.B* is calculated as distance *d* using cosine distance (default). If the distance to *template.A* is smaller, a prediction of “subclass A” is assigned to sample S. A prediction of “subclass B” is similarly performed.

### Step 3: Compute prediction confidence

Significance for the prediction is compued as a nominal p-value estimated based on a null distribution for the distance *d* to the templates generated by randomly resampling *n_A_*+*n_B_* genes from the *N* genes 1,000 times (default). This computation is performed within the data of sample S and does not use data of other samples in the test dataset. When prediction analysis is performed for multiple samples (≥2), one might want to correct the set of prediction confidence p-values for multiple hypothesis testing. In this article, we used false discovery rate (FDR)<0.05 for the criteria of high-confident prediction.

NTP can be flexibly applied to prediction of clinical disease subtypes (Example 1), cross-platform prediction of molecular disease subtype (Example 2), cross-species phenotype prediction (Example 3), and multiclass (>2 classes) prediction (Example 4) without any special optimization of analytical parameters. Furthermore, we demonstrate that NTP performs reasonably well in predicting molecular subclasses in real-world, large-scale datasets of breast cancer (Example 5). (See **Table 1** for the details of each dataset.)

**Table 1:** Datasets.

Example		No. of samples	Assay platform	Source of dataset	Reference
1. ALL vs. AML	Training set	38	HuGeneFL*	(a)	[10]
	Test set	35	HuGeneFL*	(a)	[10]
2. ER positivity in breast cancer	Training set	97	Hu25K**	(b)	[11]
	Test set	49	HuGeneFL*	(a)	[12]
3. Liver cirrhosis in human and rat	Training set	23	HG-U133plus2*	GSE6764	[13]
	Test set	12	Rat Genome 230*	GSE13747	-
4. Multiple tissue types (breast, lung, prostate, colon)	Training set	51	HG-U95A*	(a)	[14], [15]
	Test set	52	HG-U95A*	(a)	[14], [15]
5. Molecular subclasses of breast cancer	Training set	295	Stanford cDNA	(c)	[16]
	Test set 1 (“TransBig”)	198	HG-U133A*	GSE7390	[18]
	Test set 2 (“Wang”)	286	HG-U133A*	GSE2034	[19]
	Test set 3 (“Weigelt”)	53	Human WG6***	E-TABM-543	[17]

ALL: acute lymphoblastic leukemia, AML: acutr myeloid leukemia, ER: estrogen receptor, GSE: NCBI's Gene Expression Omnibus accession ID (www.ncbi.nlm.nih.gov/geo/).

E-TABM: EBI's Array Express accession ID (www.ebi.ac.uk/arrayexpress/).

(a) www.broad.mit.edu/cgi-bin/cancer/datasets.cgi.

(b) www.rii.com/publications/default.htm.

(c) microarray-pubs.stanford.edu/wound_NKI/explore.html.

All datasets used for the analysis are available as Supporting Information.

Microarrays manufactured by *Affymetrix (Santa Clara, CA), ** Agilent Technologies (Palo Alto, CA), or ***Illumina (San Diego, CA).

### Example 1. Prediction of acute lymphoblastic leukemia (ALL) and acute myeloid leukemia (AML)

**Figure 2:**
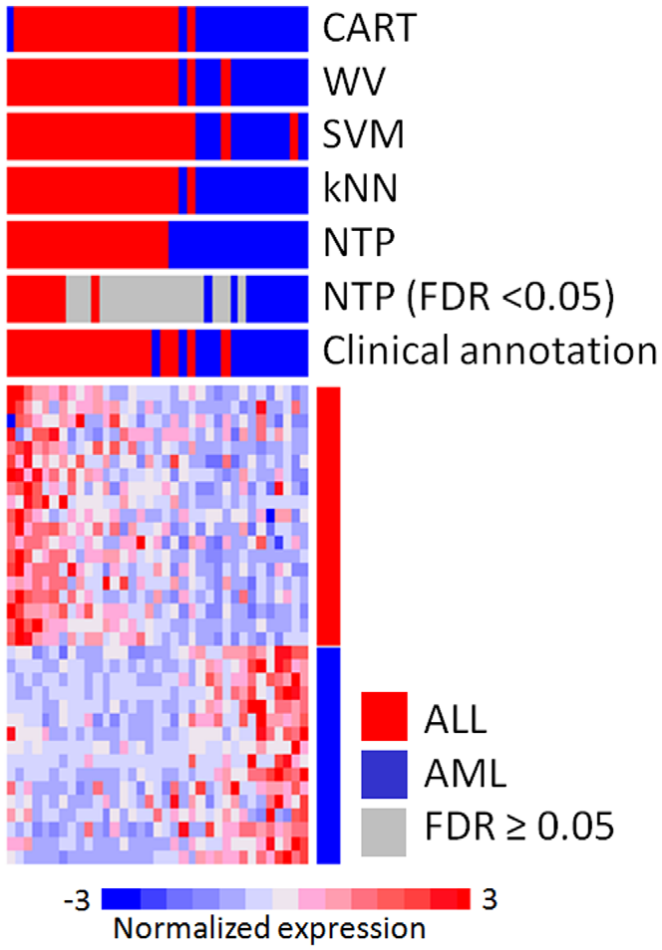
Example 1: Prediction of acute lymphoblastic leukemia (ALL) and acute myeloid leukemia (AML). Previously reported ALL-AML signature was evaluated on the test set (N = 35) employed in the study. Samples are ordered according to proximity to either of the template of ALL or AML to visualize clear or unclear expression pattern of the signature genes in each sample. Note that closer distance to template (i.e., most left or right in heatmap) does not necessarily indicate higher prediction confidence because the confidence p-value is calculated based on null distribution for the distance generated for each individual sample. CART: classification and regression tree, WV: weighted voting, SVM: support vector machine, kNN: k-nearest neighbor, FDR: false discovery rate.

First, we applied NTP for prediction of the major leukemia subtypes, ALL and AML [10] as a straightforward 2-class prediction problem in comparison with other commonly used methods: classification and regression tree (CART), weighted voting (WV), support vector machine (SVM), and k-nearest neighbor (kNN). We evaluated a 35-gene signature reported in the original publication in the test set including 35 patients (**Table 1**). Prediction error rate was comparable to other methods and even lower in the samples with high prediction confidence (FDR<0.05) (**Table 2**). Samples receiving low prediction confidence (FDR≥0.05) showed no obvious high expression of either of ALL or AML marker genes, and more number of inconsistent predictions across the different prediction methods were observed in these samples (**Figure 2** **,** **Table 3**).

**Table 2:** Summary of prediciton error rates according to prediction method.

Example	CART	WV	SVM	k-NN	NTP	NTP (FDR<0.05)
#1	9%	3%	9%	6%	11%	0%
#2	8%	14%	20%	12%	10%	5%
#3	0%	17%	0%	17%	0%	0%
#4	31%	-*	2%	6%	0%	0%
#5 (test set 1)	47%	-*	30%	47%	42%	42%
#5 (test set 2)	37%	-*	27%	44%	31%	28%
#5 (test set 3)	40%	-*	26%	23%	32%	17%

CART: classification and regression tree, WV: weighted voting, SVM: support vector machine, k-NN: k-nearest neighbor, NTP: nearest template prediction, FDR: false discovery rate.

*WV is designed only for 2-class prediciton.

In Example #5, prediction error indicates inconsistency with prediciton made with “Sorlie2003 SSP” predictor [17].

**Table 3:** Prediciton results according to prediction confidence.

Example	% samples with FDR<0.05	Error rate		Consistency across prediction methods	
		FDR<0.05	FDR≥0.05	FDR<0.05	FDR≥0.05
#1	49%	0%	22%	88%	78%
#2	88%	9%	50%	86%	17%
#3	100%	0%	-*	0%	-*
#4	100%	0%	-*	63%	-*
#5 (test set 1)	97%	42%	67%	38%	0%
#5 (test set 2)	90%	28%	64%	36%	4%
#5 (test set 3)	57%	17%	52%	50%	26%

FDR: false discovery rate.

*No sample received NTP prediciton with FDR≥0.05.

In Example #5, prediction error indicates inconsistency with prediciton made with “Sorlie2003 SSP” predictor [17].

### Example 2. Cross-platform prediction of estrogen receptor (ER) positivity in breast cancer

**Figure 3:**
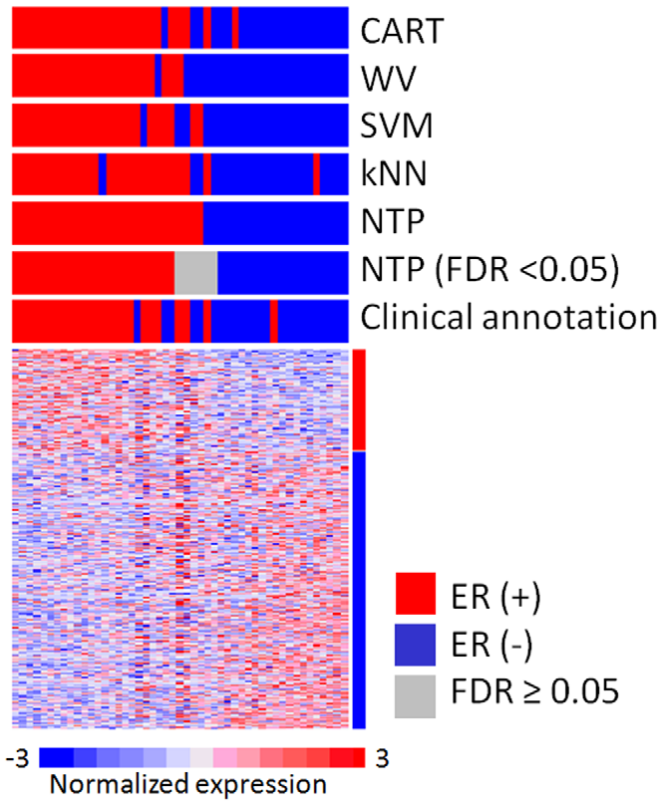
Example 2: Cross-platform prediction of estrogen receptor (ER) positivity in breast cancer. The ER signature was defined and tested on a pair of independent training (N = 97) and test (N = 49) datasets generated on different microarray platforms (see Table 1 for details). Samples are ordered according to proximity to either of the template of ER (+) or ER (−).

We next tested NTP in the setting of cross-platform prediction analysis. The ER positivity signature, which consists of 202 up-regulated and 552 down-regulated genes, was defined in the training set of 97 samples [11], and assessed on the test set of 49 samples [12] (**Table 1**, **Figure 3**). Again, prediction errors and inconsistent predictions across different methods accumulated within the samples with low prediction confidence (FDR≥0.05) (**Table 2** and **3**).

### Example 3. Cross-species prediction of liver cirrhosis between human and rat

**Figure 4:**
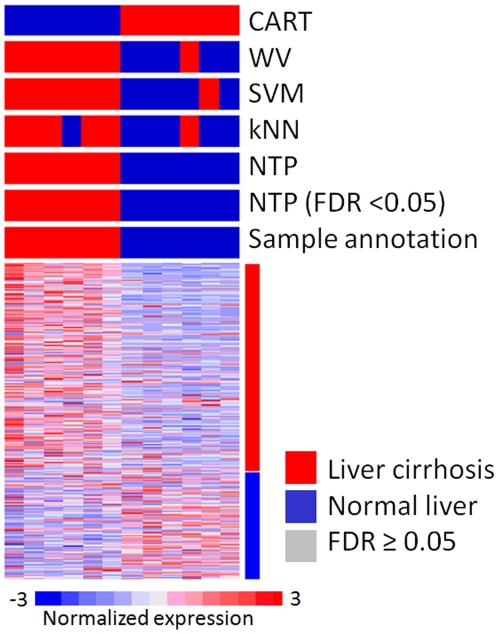
Example 3: Cross-species prediction of liver cirrhosis between human and rat. Human liver cirrhosis signature generated by comparing 13 cirrhotic and 10 normal livers was tested on rat livers with or without liver cirrhosis induced by bile duct ligation.

In this example, we evaluated NTP's prediction performance in cross-species prediction. We first defined a human liver cirrhosis signature including 801 up-regulated and 445 down-regulated genes in comparison between 13 cirrhotic and 10 normal livers from publicly available dataset [13] (**Table 1**). We then tested whether the signature was presented in another publicly available dataset of gene-expression profiles of rat liver cirrhosis induced by bile duct ligation. NTP made highly confident prediction (FDR<0.05) for all samples with no error and showed no inferiority to other methods (**Figure 4** **,** **Table 2** and **3**).

### Example 4. Prediction of multiple tissue types

**Figure 5:**
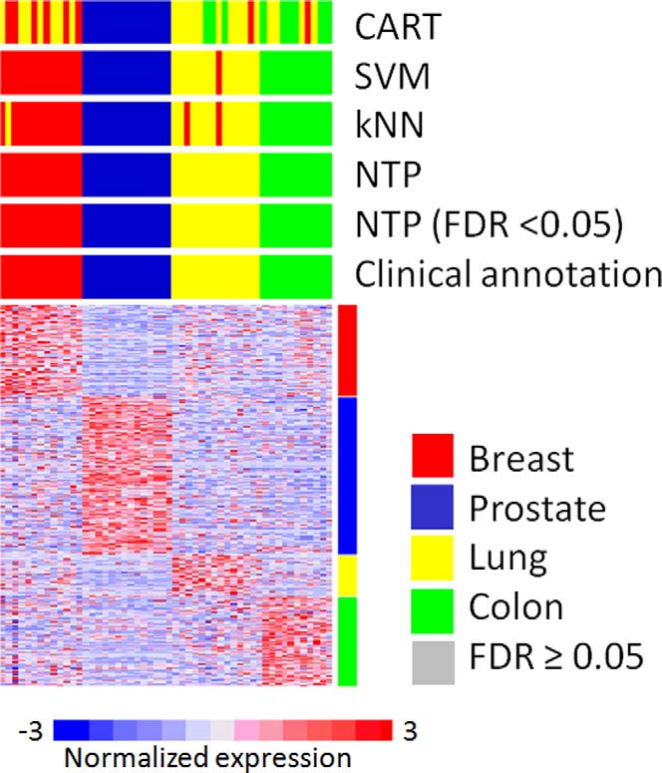
Example 4: Prediction of multiple tissue types. A signature distinguishing 4 tissue types (breast, prostate, lung, and colon) was defined in the training set (N = 51) and evaluated on the test set (N = 52). The signature was defined as a concatenation of over-expressed genes in each tissue type in comparison to the rest.

All the previous examples demonstrated the predictive performance in simple 2-class prediction. This example is to assess NTP in multi-class (>2 classes) prediction. A gene-expression signature predicting 4 different tissue types (breast, prostate, lung, and colon) was determined in the training set (N = 51) and evaluated in the test set (N = 52) (**Table 1**) [14], [15]. The signature includes 4 groups of significantly over-expressed genes in each tissue type in comparison to the rest (Breast: 388 genes, Prostate: 667 genes, lung: 174 genes, and colon: 374 genes). In this setting of multi-class prediction, NTP again demonstrated high-confident prediction (FDR<0.05) for all samples without making error (**Figure 5**, **Table 2** and **3**).

### Example 5. Prediction of breast cancer molecular subclass

**Figure 6:**
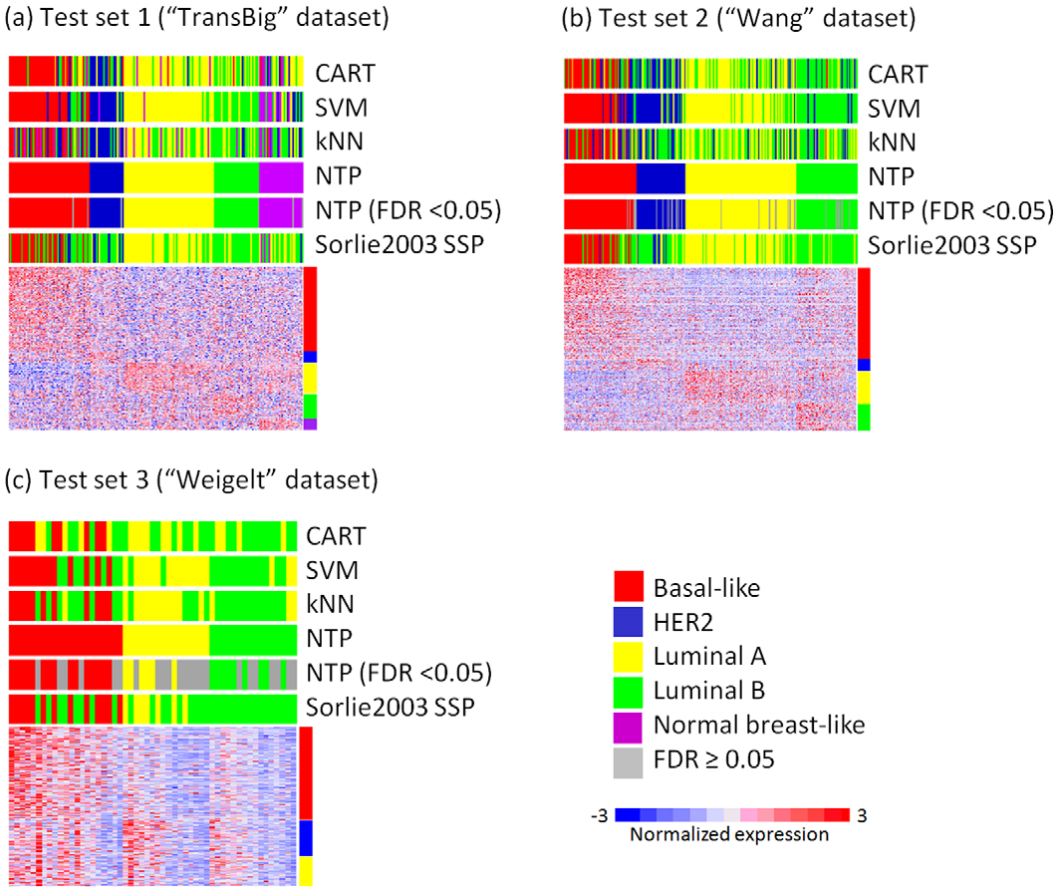
Example 5: Prediction of breast cancer molecular subclass. A signature distinguishing 5 breast cancer subclasses was defined in the training set (N = 295) and evaluated in 3 independent test sets: (a) test set 1 (“TransBig”, N = 198), (b) test set 2 (“Wang”, N = 286), and (c) test set 3 (“Weigelt”, N = 53). The signature was defined as a concatenation of over-expressed genes in each subclass in comparison to the rest.

Lastly, we analyzed multiple large scale datasets of breast cancer as an example of molecular subclass prediction in real-world clinical samples. Signature genes of the 5 subclasses (Basal-like, HER2, Luminal A, Luminal B, and Normal breast-like) were defined in the largest dataset (N = 295) [16] based on prediction made with “Sorlie2003 single sample predictor (SSP)” [17], and evaluated in 3 datasets of independent patient series generated on various microarray platforms [17]–[19] (**Table 1**). The signature consists of over-expressed genes in each subclass (Basal-like: 956 genes, HER2: 125 genes, Luminal A: 387 genes, Luminal B: 291 genes, and Normal breast-like: 134 genes). We asked whether NTP and other prediction methods could reproduce the prediction reported in the original publication (**Figure 6**). The prediction error rate was not inferior to those of other methods (**Table 2**), and consistency of prediction across the methods was higher in samples with high prediction confidence (FDR<0.05) (**Table 3**). In the original publication by Weigelt et al., 3 different SSP models were compared and yielded averaged error rates of 40%, 38%, and 38% in Test set 1, 2, and 3, respectively [17], which are comparable or even worse compared to the results of NTP (**Table 3**).

## Discussion

In gene-expression microarray analysis, signature-based approach, in contrast to single gene-based approach, has been successfully utilized for robust identification of molecular pathway activation, prediction of disease phenotype and outcome, monitoring of response to genetic or pharmacologic experimental perturbations [2], [10], [20]–[22]. These examples of success seem to be achieved particularly because the signature genes exhibit coherently similar expression pattern, and missing or failed measurements from some of the signature genes (due to malfunctioning probe, genetic polymorphism at probed site, etc.) could be compensated by rest of the genes in capturing the same trend of biological dysregulation. By focusing on such direction of expression change measured by similarly behaving multiple genes, the analysis may become less affected by difference in experimental condition and assay platform. NTP was intended to capture such direction of expression change of the signature genes, in the modeling of the templates. Our examples clearly showed that the method works reasonably well in prediction of 2 or more classes beyond different assay platforms and species without necessity of any special analysis parameter optimization. In general, no specific prediction algorithm universally outperforms others (so called no free lunch theorem), but it would worth to note that NTP showed constantly low error rate in predictions with high confidence.

In gene-expression profiling studies utilizing clinical specimens, it is frequently noticed that a certain proportion of samples do not present obvious expression pattern of the signature genes characteristic to the subclasses. In fact, we observed such samples in the middle of the heatmap in Example 1 and 2 lacking obvious high expression of ALL/AML or ER signature genes (**Figure 2** and **3**). We observed that the discordant predictions between different methods tended to accumulate within these samples, suggesting that such discordance occured due to the vague expression pattern rather than superiority of certain prediction algorithm.

The vague expression pattern may not be a problem in understanding biological nature of gene-expression signature in a set of multiple samples as long as a subset of samples presents distinct expression pattern of the signature. However, if the signature is intended to be used as a clinical diagnostic or prognostic tool, the assay and prediction need to be performed on a single patient basis. And assessment of prediction confidence is necessary to make reliable clinical decision considering any classification and prediction algorithms could assign predicted class labels even in randomly generated data. Some existing prediction methods assign prediction class label and/or prediction confidence according to relative expression of signature genes within a given collection of samples, i.e., these methods merely split the dataset into equal-sized subsets using a cut-off such as median specific to the sample collection, therefore each prediction could be changed according to which samples are included in the collection [7], [23], [24]. Weigelt et al. reported a method assigning prediction for each single patient based on correlation to “centroid” (a vector of mean expression levels of signature genes calculated for each subclass in training set) [17]. However, the criteria of “unclassifiable” sample (correlation coefficient <0.1) is arbitrary, and the performance of “centroid” might be affected when the numerical values in the vector of “centroid” overfit training set and if substantial cross-platform difference exists in test set.

In actual clinical setting, it is possible that molecular subclasses are presented in imbalanced manner across patient populations. In fact, in Example 5, some subclasses are underrepresented or even does not exist in some patient series. Even in such situation, NTP showed reasonable predictive performance constantly comparable or not inferior to other methods, supporting its usefulness as a clinical tool.

NTP is a convenient method, which allows flexible assessment of any existing gene signatures across wide variety of patient populations, assay platforms, and even species even if there is no corresponding training dataset. It will facilitate extensive preclinical evaluation of existing genomic signatures for their potential value as reliable medical diagnostics. The NTP methodology is implemented as Nearest Template Prediction module of GenePattern analysis toolkit and publicly available from www.broadinstitute.org/genepattern.

## Materials and Methods

### Data preprocessing

We utilized data sets already normalized in the respective studies. Multiple probes corresponding a single gene were summarized into a gene symbol provided by NBCI Entrez Gene database (www.ncbi.nlm.nih.gov/gene) by taking median of their signal intensities. Rat genes were converted into orthologous human genes by using a mapping table provided by the Jackson laboratory (http://www.informatics.jax.org/).

To capture “up” or “down” regulation of signature genes modeled as the template, dynamic range of measurement for each gene probe is standardized to be similar or comparable across gene probes on the microarray. Once clinical deployment platform is decided, the assay is assumed to be calibrated to generate reproducible measurements and a set of reference samples is run to test its validity. This reference dataset will allow to calculate sample-wise mean and sample standard deviation for each gene probe, which can be used for the standardization of each single patient's data obtained in actual clinical practice. NTP can take vectors of the mean and sample standard deviation provided separately from input dataset for the standardization (supported in the R code packaged in the GenePattern NTP module). Alternatively, when such reference dataset does not exist and a research-level dataset is the only available material, NTP also can use the input dataset to calculate the mean and sample standard deviation for the standardization (current default of the GenePattern module).

### Methodology of Nearest Template Prediction (NTP)

#### (i) Representative expression pattern of the signature genes (template)

Suppose the case of prediction of 2 subclasses, A and B, and the signature to be tested includes *n_A_* and *n_B_* over-expressed marker genes in subclass A and B, respectively. The template of subclass A (*template.A*) is defined as a vector containing *n_A_*+*n_B_* elements: first *n_A_* elements correspond over-expressed genes in subclass A (*marker.A*), and next *n_B_* elements correspond over-expressed genes in subclass B (*marker.B*). To model representative expression pattern of subclass A, i.e., over-expression of *marker.A* and under-expressed of *marker.B*, a value of 1 is assigned to each of the *n_A_ marker.A* genes, and a value of −1 to each of the *n_B_ marker.B* genes. The template of subclass B (*template.B*) is similarly defined. In case of prediction of more than 2 subclasses, a value of 1 is assigned to subclass A marker genes and a value of 0 is assigned to marker genes of the rest of subclasses. Numerical values like *t*-statistic, signal-to-noise ratio, fold change, or p-value, which is often reported together with list of signature genes, can be used to weigh each gene to incorporate information of its relative importance in the signature.

#### (ii) Nearest template to assign a prediction label to a test sample

For a test sample, actual expression levels of the *n_A_*+*n_B_* signature genes are extracted from *N* genes measured in the microarray assay (*N*≥*n_A_*+*n_B_*) to create a vector with *n_A_*+*n_B_* elements (*sample.signature*). Proximity of *sample.signature* to *template.A* or *template.B* is computed as distance *d* using cosine distance (default) or Pearson correlation coefficient. Any other distance measure can be used. If *d* to *template.A* is smaller than that to *template.B*, a prediction label of subclass A is assigned to the test sample, and a prediction of subclass B is similarly performed.

#### (iii) Prediction confidence (p-value)

For the single test sample, significance of the proximity to template is estimated as a nominal p-value based on a null distribution for the distance *d* generated by randomly resampling *n_A_*+*n_B_* genes from the *N* genes multiple times (default is 1,000 times). Here, the null hypothesis is that the signature genes show similar expression pattern to neither of the templates, and the alternative hypothesis is that the signature genes show similar expression pattern to any one of the templates. A nominal p-value is computed based on the rank of actual *d* in the null distribution. In case the prediction is made for a set of multiple samples (≥2), user can choose to correct the p-values for multiple hypothesis testing using either false discovery rate (FDR) [25] or Bonferroni correction.

### Prediction analysis in each example

In **Example 1**, the list of signature genes reported in the original publication was directly used for NTP, including 19 up-regulated (GOLUB_ALL_VS_AML_UP) and 16 down-regulated (GOLUB_ALL_VS_AML_DN) genes in ALL compared to AML samples deposited in Molecular Signature Database (MSigDB) (http://www.broadinstitute.org/msigdb/). The signature genes In **Example 2∼5** were defined by random permutation-based *t*-test implemented in Comparative Marker Selection module of GenePattern analysis toolkit [26] (www.broadinstitute.org/genepattern) with a significance threshold of FDR<0.05 (**Example 2∼4**) or FDR<0.001 (**Example 5**). In multi-class (>2 classes) prediction (**Example 4 and 5**), the signature was defined as a concatenation of over-expressed genes in each class in comparison to the rest. Genes significantly over-expressed in multiple classes were removed from the signature. In **Example 4**, the dataset including 4 tissue types (breast, prostate, lung, and colon) was randomly split into training (N = 51) and test (N = 52) sets using Split Dataset Train Test module of GenePattern.

### Prediction analysis with other methods

Additional prediction analyses were performed within the same signature gene space by using CART, WeightedVoting, SVM, and KNN modules implemented in GenePattern with default analysis parameters. For cross-platform (Example 2 and 5) and cross-species (Example 3) predictions, each gene's expression level was standardized using its sample-wise mean and sample standard deviation in each dataset to adjust range of gene expression level between training and test datasets. All datasets and class labels used for the analysis are publicly available at http://www.broadinstitute.org/cgi-bin/cancer/datasets.cgi.
